# Supplementary material for: ‘This Adds Another Perspective’: Qualitative Descriptive Study Evaluating Simulation-Based Training for Health Care Assistants, to Enhance the Quality of Care in Nursing Homes
Source: Int J Environ Res Public Health. 2021 Apr 10;18(8):3995. doi: 10.3390/ijerph18083995 (PMC8069740; doi:10.3390/ijerph18083995)
Supplement: Supplementary file 1 [file ijerph-18-03995-s001.pdf]

<u>Supplementary File 1:</u>

Manager in-depth interview topic guide

The topic guide will be used flexibly.

Please tell me about your role in as a manager

(If interview is conducted after training:) Have you noticed any differences among the HCAs that you manage, since they did the training? If so, please tell us about it

What are your perceptions of the training needs of HCAs?

What are your perceptions of how HCAs could best be supported?

What are the barriers/facilitators to HCAs' training and development?

Supplementary File 2:

Mentor/clinical lead/supervisor in-depth interview topic guide

The topic guide will be used flexibly.

Please tell me about your role in supporting HCAs

(If interview is conducted after training:) Have you noticed any differences among the HCAs that you support, since they did the training? If so, please tell us about it

What are your perceptions of the training needs of HCAs?

What are your perceptions of how HCAs could best be supported?

What are the barriers/facilitators to HCAs' training and development?

Supplementary File 3:
Topic Guide for Phase 1 HCA In-Depth Interviews: pre-training
(the topic guide will be used flexibly)

- A bit about you:
    - Route to/reasons for working in residential care
    - How long have you been working in this care home? In residential care overall?

- Describe a typical shift

- Views/feelings about residential care work in general
    - Things that you find rewarding & why
        - Specific example/s
    - Things that you find challenging & why
        - Specific example/s
    - How important or unimportant is it to you, to feel valued in your job? Do you feel valued? By whom/how?
    - Do you see yourself working in care work in 6 months' time? 5 years' time? Why/why not?
    - If yes… In this home or another home?

- Self-identified training / support / development / other needs: Is there anything that could help you to do your job better? (describe what/how)
    - Feel prepared to deal with circumstances you come cross in your job?
    - Prompt: training needs? How/why could this help?
    - Impact on your feelings about continuing working in residential care homes?

- Experiences of training received in the past
    - What were the training sessions like, how delivered?
    - Did it affect your work? How? Relevant? Helpful?
    - How did the training affect your feelings about continuing working in residential care homes?
    - Some people tend to avoid non-compulsory training, whereas others seek it out – can you describe where you fit along this continuum? What affects this?

- Do you care for residents with dementia? If so…
    - Can you describe what they are like; how do they differ from people without dementia?
    - Describe what it is like to care for people with dementia

- Probe: positive & negative aspects – specific examples, e.g. last time this happened
- Confidence in this area? Anything that could help?

- As part of your work, do you liaise with health/medical professionals within/outside the care home, on residents' behalf? By this I mean – for example – GPs, doctors, emergency services (ambulance/A+E), nurses or specialists involved in your residents' medical care.
    - If yes…

    What is this like? Please describe the last time this happened. Describe a time when you felt this communication/interaction went well; a time when it didn't go so well. *I do not need details of people or specific places but rather the chain of events and how you felt about it.*
    - If no… Might this happen in future? Who handles these communications, in this care home? Feelings about communicating with medical/health professionals
    - Confidence in this area? Anything that could help?

At the end of the interview, a questionnaire (the Humanised Care Assessment Tool, HCAT) was piloted with HCAs and brief qualitative feedback sought on it, from HCAs and mentors. Piloting the HCAT was a subsidiary objective of the study, and so findings are not presented in this paper.

<u>Supplementary File 4:</u>
Phase 2 interviews: post training
The topic guide will be used flexibly.

- In general, how have things been at work, in the last month or so?

- Views/feelings about care work
  - Things that you find rewarding & why
    - Specific example/s
  - Things that you find challenging & why
    - Specific example/s
  - How important or unimportant is it to you, to feel valued in your job? Do you feel valued? By whom/how?
  - Do you see yourself working in care work in 6 months' time? 5 years' time? Why/why not?
  - If yes… In this home or another home?

**After Walk through Dementia training:**	**After simulated SBAR training:**
In the past month or so, in general, how have residents with dementia been?<br>What has it been like caring for them?<br><br>Has it got better/worse/no change since the training?<br>Prompts:<br>• your ability to relate to / empathise with residents with dementia?<br>• Frustrations/challenging situations?<br>• Confidence in caring for people with dementia?<br>• Feeling valued?<br>• Feelings about staying within the care home sector<br><br>Reflections/feedback/suggestions on the **A Walk through Dementia** training	Have you liaised with medical/health professionals in the past month or so, about people in your care?<br>If so… What was it like/ how did it go?<br>Has it got better/worse/no change since the training?<br>Prompts: In terms of…<br>• Communicating with healthcare professionals and colleagues, about residents' health/medical situations?<br>• Your ability to relate to / empathise with colleagues?<br>• Frustrations/challenging situations?<br>• Feeling valued?<br>• Feelings about staying within the care home sector<br><br>Reflections/feedback/suggestions on the **simulated SBAR** training

Supplementary File 5: Feedback form

We are interested in your views of the training you received today. What you tell us will help us to improve the training.

How relevant was the training to your work?<br>*Please circle an answer:*	Very relevant	Relevant	Not relevant	Not relevant at all

Please tell us why you chose that answer:

How helpful or unhelpful did you find the training?<br>*Please circle an answer:*	Very helpful	Helpful	Not helpful	Not helpful at all

Please tell us why you chose that answer:

How enjoyable was the training?<br>*Please circle an answer:*	I really liked it	I liked it	I did not like it much	I did not like it at all

Please tell us what you liked or disliked:

Was the duration of today's training session…		Too short	About right	Too long

Have you done training on SBAR/dementia[1] before?		Yes	No	I can't remember
If YES: Compared with the training you did before, was today's training:				
	Much more relevant	A bit more relevant	A bit less relevant	Much less relevant
	Much more helpful	A bit more helpful	A bit less helpful	A lot less helpful
	Much easier to understand	A bit easier to understand	A bit more difficult to understand	A lot more difficult to understand
	Much more enjoyable	A bit more enjoyable	A bit less enjoyable	Much less enjoyable

How will you use what you learnt today?

We'd like your suggestions to help us improve the training in future. Please tell us a few things that could make the training better:

[1] Deleted as applicable, i.e. after SBAR simulation training participants were asked whether they had been trained on SBAR before; after AWTD training they were asked whether they had been trained on dementia before.

Thank you for completing this questionnaire!
